# A Transdisciplinary Complex Adaptive Systems (T-CAS) Approach to Developing a National School-Based Culture of Prevention for Health Improvement: the School Health Research Network (SHRN) in Wales

**DOI:** 10.1007/s11121-018-0969-3

**Published:** 2018-12-08

**Authors:** Simon Murphy, Hannah Littlecott, Gillian Hewitt, Sarah MacDonald, Joan Roberts, Julie Bishop, Chris Roberts, Richard Thurston, Alexa Bishop, Laurence Moore, Graham Moore

**Affiliations:** 1 Centre for the Development and Evaluation of Complex Interventions for Public Health Improvement (DECIPHer), School of Social Sciences, Cardiff University, 1-3 Museum Place, Cardiff, CF10 3BD UK; 2 Health Improvement Division, Public Health Wales, Cardiff, UK; 3 Knowledge and Analytical Services, Welsh Government, Cardiff, UK; 4 Cancer Research UK, Cardiff, UK; 5 MRC/CSO Social and Public Health Sciences Unit, University of Glasgow, Glasgow, UK

**Keywords:** School networks, Health improvement, Transdisciplinary, Complex adaptive systems

## Abstract

The paper reflects on a transdisciplinary complex adaptive systems (T-CAS) approach to the development of a school health research network (SHRN) in Wales for a national culture of prevention for health improvement in schools. A T-CAS approach focuses on key stages and activities within a continuous network cycle to facilitate systems level change. The theory highlights the importance of establishing transdisciplinary strategic partnerships to identify and develop opportunities for system reorientation. Investment in and the linking of resources develops the capacity for key social agents to take advantage of disruption points in the re-orientated system, and engagement activities develop the network to facilitate new social interactions and opportunities for transdisciplinary activities. A focus on transdisciplinary action research to co-produce interventions, generate research evidence and inform policy and practice is shown to play an important part in developing new normative processes that act to self-regulate the emerging system. Finally, the provision of reciprocal network benefits provides critical feedback loops that stabilise the emerging adaptive system and promote the network cycle. SHRN is shown to have embedded itself in the system by securing sustainability funding from health and education, a key role in national and regional planning and recruiting every eligible school to the network. It has begun to reorient the system to one of evidence generation (56 research studies co-produced) and opportunities for data-led practice at multiple levels. Further capacity development will be required to capitalise on these. The advantages of a complex systems approach to address barriers to change and the transferability of a T-CAS network approach across settings and cultures are highlighted.

## Introduction

Adolescence is a period characterised by rapid biological maturation and significant behavioural and social change. Hence, it is an important window of opportunity for early intervention to promote health across the life course (Viner et al. 2015). Schools are an important setting for young people’s health improvement, and there is growing evidence of ‘school effects’ on health (Bonell et al. 2013). However, the array of demands schools face mean their willingness to devote time to student health and wellbeing is variable, compounded by the notion of a zero-sum game between promoting academic attainment and promoting health (Bonell et al. 2014). The widespread failures of school health researchers to consider impacts of health interventions on schools’ core educational business has led to this assumption going largely unchallenged and likely contributed to intervention implementation failure (Langford et al. 2014).

However, even where schools are supportive of health improvement, there are substantial challenges to improving health and wellbeing in the school setting (Langford et al. 2016). Crucially, there has been a failure to integrate academic, policy, practice and public communities to co-produce school health improvement research and build in processes to understand intervention congruence with existing systems and structures, and hence their sustainability. Co-production regards non-academics as active agents in research and strives for equal, mutually beneficial and reciprocal relationships which value public, practitioner and policy-maker knowledge and experience to the same degree as academic knowledge (Heaton et al. 2016). This lack of integration and co-production means that a number of factors that impede school-based health improvement research persist in the UK. (Flinders et al. 2016).

Responding to a similar situation in healthcare, UK Clinical Research Network initiatives have facilitated cultural change for evidence generation and practice. These networks highlight the value of investing in infrastructures that support research capacity development and foster a culture of practitioner-led enquiry. Critically, they have adopted transdisciplinary approaches to integrate academic, policy-maker and practitioner communities to generate and translate policy- and practice-relevant research for improved health outcomes.

Transdisciplinary approaches emphasise innovation in order to generate and translate scientific evidence that can be practically applied to address societal problems (Stokols et al. 2013). Such innovation requires sustained collaboration between academic disciplines, practitioners, policy-makers and the public, whose diversity of knowledge, experience and perspectives maximise the potential for scientific and translational innovation and impact (Stokols et al. 2013). Transdisciplinary action research (TAR) cycles are a process for cultivating and sustaining such collaborations in order to achieve shared goals by linking three types of collaboration: (1) transdisciplinary scientific collaboration, (2) collaborations among researchers and community practitioners and (3) inter-sectoral partnerships for designing and implementing public policies (Stokols 2006; Stokols et al. 2013).

Potential exists to transfer such a cycle to the school setting, but unlike the clinical research networks described above, the challenge arises of bridging two distinct policy areas (education and health) and crucially, centres on practices not immediately embraced by all as directly relevant to ‘core business’. Collaborations which bring together health researchers with education practitioners are relatively rare, but some international models exist (Cameron et al. 2007; Riley et al. 2011). TAR holds promise, but gaps in our understanding remain, including the challenges associated with a transdisciplinary network spanning multiple sectors and ecological levels. Previous research has identified factors which may facilitate collaboration within TAR networks, such as the need for joint aims and objectives, extended time periods and shared language (Littlecott et al. 2017; Spoth and Greenberg 2011), but these studies have neglected to take into account the functioning of the complex systems within which collaborations exist.

Systems thinking conceptualises the interrelationships between parts or components of a system (such as a school, or the broader education system in which it is nested) and their relationships with the system as a whole (Trochim et al. 2006). Schools can be conceptualised as complex adaptive systems (CASs) (Keshavarz et al. 2010), a dynamic network of diverse agents, constantly acting and reacting to other agents’ behaviour. System functioning emerges from these interactions, in turn influencing individuals’ behaviour in a context-dependent and inconsistent manner (Keshavarz et al. 2010). CASs have a propensity towards self-organisation, with order emerging through collective actions of agents within the system, rather than central planning. They are ‘adaptive’ in that they are constantly evolving in line with wider changes to surrounding systems. Where a relatively stable ‘attractor state’ is disrupted by internal or external changes, agents work to return the system to a new form of order. The functioning of CAS is largely sustained by feedback loops, with feedback on the impacts of a way of working acting as inputs for subsequent actions; these may be positive-reinforcing, leading to continuation of a way of working, or negative-balancing, leading to discontinuation. Schools operate and adapt in synergistic exchange with other external systems, exercising autonomy over their own ways of working, within limits set by the educational ‘supra-systems’, and broader political and economic supra-systems, in which they are nested (Keshavarz et al. 2010).

Systems such as schools are comprised of (i) activity settings (e.g. classrooms, parent-teacher meetings), (ii) social networks that link these settings and (iii) time (Hawe et al. 2009). Interventions represent an attempt to change existing school dynamics in order to activate the health-enhancing (and limit health harming) potentials of school systems. Introducing change in a CAS creates disruption, forcing agents to work collectively to restore the system to order, through assimilating a new way of working into the everyday functioning of the system, or washing it out (Hawe et al. 2009).

Engagement with how systems work before attempting to change them, as advocated from a CAS perspective, is a key characteristic of the School Health Research Network (SHRN). SHRN (Hewitt et al. 2018) was launched in Wales in 2013 as a strategic partnership between Cardiff University, Welsh Government, Public Health Wales (part of the National Health Service in Wales) and Cancer Research UK (a research-focused charity). Network members are schools serving mainstream students aged 11 to 18 years. The network is led by a multidisciplinary research team in the Centre for the Development and Evaluation of Complex Interventions for Public Health Improvement (DECIPHer) at Cardiff University, a UK Clinical Research Collaboration (UKCRC) Centre of Public Health Research Excellence. SHRN utilises a TAR network cycle approach, implemented within a CAS perspective. An integrative transdisciplinary complex adaptive systems (T-CAS) approach was developed to guide network development, which has successfully:

- Established new cross-sector stakeholder partnerships at multiple levels
- Embedded network activity within national and local policy frameworks
- Built a national data infrastructure with biennial collection of student and school-level health and wellbeing data
- Established a programme of school engagement activities to secure membership of 212 (100%) secondary schools in Wales
- Co-produced scientific evidence and established a new data-led planning system
- Developed research capacity to generate evidence and support professional practice
- Secured resources from multiple stakeholders for long term sustainability

This paper describes and reflects on the T-CAS network cycle, which has initiated systems change towards a culture of prevention for health and wellbeing in the school system. It draws on complex systems thinking to explore each element of the T-CAS network cycle, highlighting the key system mechanisms to be triggered for systems change. The activities associated with each stage of the T-CAS approach are highlighted, along with the data used to understand network development and assess progress across its first three developmental cycles (2013 to 2018). Finally, the transferability of a T-CAS network across national settings and cultures is considered.

## A T-CAS Network Cycle

**Fig. 1:**
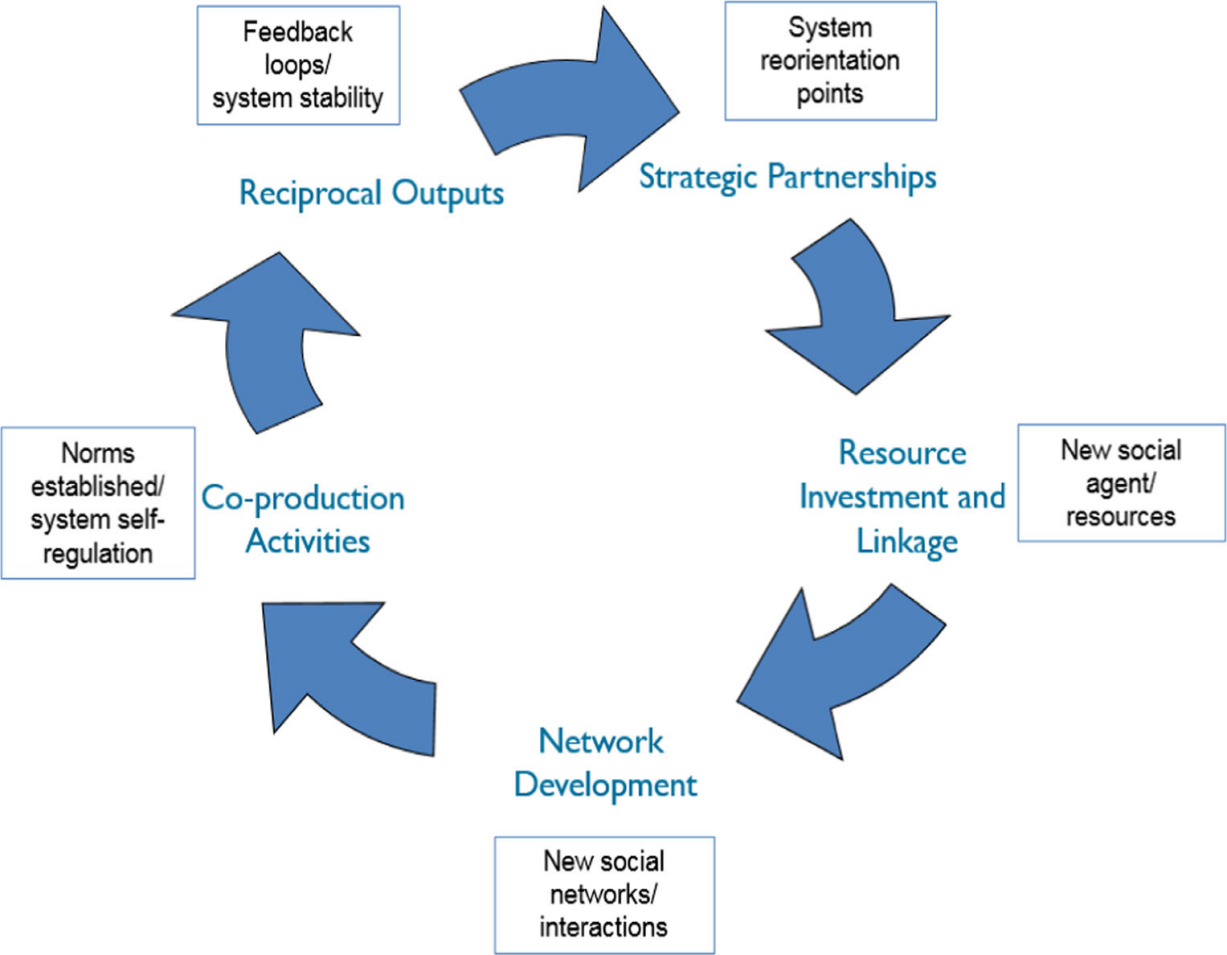
A transdisciplinary complex adaptive systems (T-CAS) network cycle^1^. ^1^The figure represents a system disruption via a Transdisciplinary Action Research Network Cycle approach. System change processes (boxed) correspond to the stages of the cycle where they are likely to be most dominant

The five stages of SHRN’s continuous T-CAS network cycle, which represents a disruption to the functioning of the school health system in Wales, are shown in Fig. 1. The disruption triggers T-CAS change processes, which sustain the cycle. Whilst these change processes happen concomitantly, the dominant system change process associated with each stage of the cycle is illustrated. Table 1 meanwhile highlights the system facilitators that have supported SHRN and the key activities associated with each T-CAS stage across the three developmental cycles to date, with the 2018 cycle currently being implemented. The following sections elaborate on each stage of the T-CAS approach underlying SHRN, how they align with system change principles and assess the network’s progress towards an enhanced culture of health improvement in schools in Wales.

**Table 1:** SHRN development: context facilitators, T-CAS activities and impacts 2013 to 2018

	Context facilitators	Cycle 1 activities 2013/2014	Cycle 2 activities 2015/2016	Cycle 3 activities 2017/2018 to date	System impacts
Strategic partnerships Goal: To develop and exploit opportunities for system reorientation	PHIRN partnership 2005. Policy opportunities—Curriculum Review 2015, Well-being of Future Generations Act 2015, Violence against Women, Domestic Abuse and Sexual Violence (Wales) Act 2015, Estyn Common Inspection Framework.	Establishment of strategic advisory board with 8 partners. SHRN representation on 2 national bodies- Education Settings Board, Tobacco Control Board	Expansion of board to 11 partners. SHRN representation on 3 national bodies - Obesity Strategy Board added.	Expansion of board to 12 partners. SHRN representation on 5 national bodies—curriculum for Wales Partnership, Sex and Relationships Education Expert Panel added.	Increases in number of cross policy and national/regional partners. SHRN representation on increased number of cross policy strategy groups. SHRN now embedded in vertical and horizontal national planning and monitoring systems with increased opportunities for system re-orientation.
Resource investment and linkage Goal: To develop social agents and resources to facilitate system change	HBSC funding 1985. DECIPHer funding 2010.	MRC and HBSC survey funds. 3 core staff - Network manager, Survey manager, Administrator. 2 DECIPHer short courses (78 attendees). 2 new PhDs and 1 principal investigator.	HCRW and PHW funds. 3 core staff. 6 DECIPHer short courses (144 attendees), Research for practice (30 attendees). 4 new PhDs and 2 principal investigators.	HCRW, PHW, Education and Health Cabinet Secretaries and HBSC funds. 4 core staff - Welsh Government funded data analyst added. 7 DECIPHer short courses (109 attendees). 6 new PhDs, and2 principal investigators.	Funding secured from both health and education. SHRN sustainability now embedded in horizontal cross policy resource infrastructure. Research capacity activities have developed social agents in academia. This has led to an increase in co-production activities and reciprocal outputs. Future cycle goals will focus on school level capacity development to capitalise on data led practice opportunities.
Network development Goal: To promote new networks and social interactions for partnership opportunities	WNHSS 1999.	30% (*n* = 69) schools and 8151 pupils. 1 national network event (22 attendees) and termly newsletters. 1 webinar—whole school approach. Academic practice seminar—suicide and self harm.	53% (115) schools and 35,071 pupils completed survey. 5 national network events (106 attendees) and termly newsletters. 7 webinars, e.g., breakfast and academic performance. Academic practice seminar—informing policy and influencing practice.	100% (212) schools and 112,045 pupils completed survey. 6 national network events (272 attendees) and termly newsletters. 3 webinars e.g. sex and relationships education. Academic Practice seminar – Gambling. 7 pupil engagement events. Regional engagement activities.	Total school system diffusion secured and over 60% pupil participation. New system network firmly established. An increase in number and reach of networking events has facilitated partnership opportunities that have resulted in co production for evidence generation and identified innovation in data led practice.
Co-production activities Goal: To establish new system norms and ways of working	PHIRN 2005. DECIPHer 2010.	5 adopted and 2 funded studies.	33 adopted and 20 funded cumulative studies.	56 adopted and 32 funded cumulative studies.	New system norms and ways of working for evidence generation firmly established. Future goals will focus on facilitating evidence translation.
Reciprocal outputs Goal: To provide benefits for multiple stakeholders to promote emergent system stability		67 reports provided to schools.	87 reports provided to schools. Report provided to WNHSS. 7 academic papers e.g. e-cigarette use. 5 lay summaries e.g. school smoking policies. Regional reports piloted.	193 reports provided to schools. Report provided to WNHSS. 5 academic papers e.g. health improvement and attainment. 3 lay summaries, e.g. school culture and health inequalities. Provision of data for Welsh Government’s Children and Young People’s Wellbeing Monitor for Wales, Well-being of Future Generations Act indicators and Active Healthy Kids Wales Report Card. 21 Regional reports for Well-being of Future Generations Act indicators.	Year-on-year increase in number and system reach of reciprocal outputs. SHRN data provision now across policy areas (horizontal) and national, regional and local levels of the education system (vertical). A new system of data-led practice opportunities has been successfully established.

Cancer Research UK (CRUK), Centre for the Development and Evaluation of Complex Interventions for Public Health Improvement (DECIPHer), Health Behaviour in School Children survey (HBSC), Health and Care Research Wales (HCRW), Medical Research Council (MRC), Public Health Improvement Research Network (PHIRN), Public Health Wales (PHW), Wales Institute of Social and Economic Research Data and Methods (WISERD) Welsh Network of Healthy Schools Schemes (WNHSS)

## Strategic Partnerships for System Reorientation

The creation of strategic partnerships working towards mutual agendas provides opportunities for system re-orientation and subsequent disruption (Hawe et al. 2009). If these strategic partnerships adequately engage with schools’ core business, this disruption has the potential to lead to system reorganisation to assimilate SHRN into the everyday functioning of the Welsh school system.

Broad horizontal and hierarchical representation is required for successful partnerships (Carey and Crammond 2015), particularly where they span policy areas that may traditionally be siloed (Bonell et al. 2014). Establishing representative network governance, inclusive communication and relationship building among hierarchical and horizontal groups of stakeholders and the development of common goals (Littlecott et al. 2017), in line with schools’ core business, has been crucial for the success of SHRN.

Hierarchical and horizontal partnerships in SHRN are operationalised through its governance structure, an Advisory Board meets biannually to provide strategic guidance and identify common programs of work. This ensures that subsequent stages of the network cycle are aligned with emerging partnership agendas. Horizontal membership spans health and educational policy makers and research staff from national government, Public Health Wales, the education inspectorate and national third sector representation. Hierarchical representation comes from WNHSS and network schools to ensure local participation in the development of shared agendas and the implementation of policy legislation. Academic membership of the Board draws on the multidisciplinary research expertise of DECIPHer and the Welsh Institute of Social and Educational Research in Wales, whilst cross national academic representation ensures consideration of potential cross cultural network translation. DECIPHer, having established a Public Health Improvement Research Network (PHIRN) in 2005, has been able to bring significant expertise and stable, long-term partnerships.

Strategic links between board members and other structures enable integration of SHRN into other relevant systems. Table 1 shows that across the three developmental cycles, SHRN has increased its representation from two to five national bodies via stakeholder invitations that recognised its increasing importance in system functioning. This has facilitated the types of interaction between and within different systems that are required for systems change (Keshavarz et al. 2010).

As well as developing comprehensive hierarchical and horizontal structures, it is also important to consider the political context of the system that might influence mutual agendas (Asthana et al. 2002). SHRN has done this by proactively aligning its agenda and activities with new policies that are relevant to schools, thus working with the system to achieve change by aligning with schools’ core business and harnessing and developing policy opportunities to take advantage of emerging disruption points within the system (Hawe et al. 2009; Keshavarz et al. 2010). SHRN provided responses to policy proposals at the consultation stage, highlighting its potential role in monitoring and evaluation and, once enacted in law, developed strategic partnerships with those responsible for their implementation. Consequently, across cycles, Advisory Board membership grew from eight organisations to 12 and new areas of collaboration established with, for example, those responsible for children’s rights and curriculum delivery.

System facilitators have included the Well-being of Future Generations (Wales) Act 2105, which established a requirement for public bodies to set and act on wellbeing objectives, and the Violence against Women, Domestic Abuse and Sexual Violence (Wales) Act 2015, which requires schools to monitor and report violence against women and girls. The most important opportunity, however, has been a school curriculum review, which now requires schools to address wellbeing as one of six main areas of curriculum delivery (Donaldson 2015).

These policy changes prioritised health within the education system and enhanced its alignment with school activities, thereby creating fertile ground to subvert existing mechanisms which may have filtered out information about health and undermined partnerships relevant to a preventive agenda (Hawe et al. 2009; Keshavarz et al. 2010). In particular, this supported resource investment, with SHRN able to secure resource investment from the Cabinet Secretaries for Education and Health in Welsh Government for policy monitoring via its data infrastructure.

## Resource Investment and Linkage for New Social Agents and Resources

Given the lack of a similar funding framework to that utilised by Clinical Research Networks, SHRN has capitalised upon its partnerships to develop and link existing resources. Provision of resources, such as knowledge, skills and finance, for the successful delivery of whole system interventions is well recognised (Gugglberger and Dur 2011) and highlights a need for capacity building at all levels to change system functioning. Such capacity building represents a major disruption to the existing system and is instrumental in preventing reorganisation back to the status quo. Instead, it progresses the system to an improved attractor state, which incorporates SHRN into everyday system functioning.

Developing social agents for change has focussed on increasing research methodologist capacity and evidence informed practitioner capacity. DECIPHer provided a strong base of transdisciplinary research capacity to promote T-CAS cycle activity, including researchers with expertise in developing and evaluating complex interventions and systems approaches to prevention and implementation science and a young peoples’ advisory board. This saw a growth in transdisciplinary studentships embedded in SHRN from 2 to 12 across the three cycles and the development of 5 new lead investigators for co-produced research studies. DECIPHer also supports capacity development through its short courses, which are promoted to both academics and practitioners and provide a mature and supportive research context for SHRN. As the network has grown, the number of courses and their uptake have increased across the developmental cycle, from 2 courses in cycle one with 78 attendees, through 6 courses with 144 attendees in cycle two, to 7 planned courses in the current cycle and 109 attendees to date.

Development of evidence informed practitioners, however, is less well developed. In 2015, SHRN piloted research literacy training for 30 public health practitioners in schools and found a relatively low base of research competency and understanding. Practitioners highlighted the need for a stronger relationship and an open line of communication with researchers to advice on how to interpret evidence and evaluate innovation. This contrasts sharply with clinical research networks where research training forms an integral part of professional education (Shepherd 2007) and will require significant investment in future implementation cycles. SHRN is also promoting evidence-informed practice by providing research briefs and webinars (see next section) and through its data infrastructure (see ‘Reciprocal Outputs for Feedback Loops and System Stability’ section), which produces timely data to support intervention development and health planning and policy monitoring at multiple system levels. More fundamental capacity development work is needed, however, to develop teacher training and continuous professional development to ensure full utilisation of these resources.

TAR networks are facilitated by linking existing resources and social agents (Hakansson and Ford 2002). Key to this in SHRN has been the development of network spanner roles (Burt 2004; Hawe and Ghali 2008). Structural hole theory emphasises the importance of such brokerage roles to enhance knowledge exchange between stakeholders within or between complex systems (Burt 2004; Hawe and Ghali 2008). Network spanner roles are embodied in the Advisory Board members, whose remit is to enhance synchronicity and seek to link resources for value-added activity. For example, network spanners provided the opportunity to establish the SHRN data infrastructure as part of the international 2013 Health Behaviour in School-Aged Children (HBSC) survey (Inchley et al. 2016) in Wales, at no additional cost. This capitalised on an opportunity for adding value through system reorganisation and developing partnerships. For the 2017 HBSC survey, the SHRN data infrastructure has provided a reciprocal benefit for Welsh Government as the HBSC survey can now be embedded within SHRN data collection, thereby reducing costs and facilitating recruitment and response rates.

Another crucial network-spanning role is the network manager who has responsibility for school recruitment and policy and practice engagement. An effective spanning role required expertise and experience as a practitioner in both health and education and experience of both research and practice to be credible to all SHRN partners. Indeed, the presence of effective brokers to bridge structural holes has been shown to be more important than having a network with a high density, or a high percentage of potential relationships being present (Burt 2004; Hawe and Ghali 2008; Heng et al. 2005). The network manager plays a vital network-spanning role in aligning the aims of the network with academics and practitioners and engaging schools through network activities. An additional spanning role has been secured in the most recent SHRN cycle, with Welsh Government investment in an analyst to facilitate reciprocal outputs.

Developing capacity and utilising network spanners create system disruption by improving information flow and resource linkage across activity settings within the school system but also requires the development of wider social networks for cultural change (Hawe 2015; Hawe et al. 2009).

## Network Development to Build New Social Networks and Interactions

New social networks have been facilitated through phased school recruitment and knowledge translation activities at different levels of the school health system. Schools across Wales have been recruited across three developmental cycles. In 2013/2014, 30% (*n* = 69) and in 2015/2016, 53% (*n* = 115) of schools joined the network, with recruited schools at each phase representative in terms of population school size, deprivation and geographical location. In 2017/2018, the network was successful in recruiting 100% (*n* = 212) of secondary schools in Wales and, to date, no schools have left the network. This translates to a growth in participating pupils from 8151 in 2013 to 112,045 in 2017.

The key engagement activity at the school level has been provision of tailored Student Health and Wellbeing Reports to schools that participate in the network’s biennial student health survey (see ‘Reciprocal Outputs for Feedback Loops and System Stability’ section below). In 2013, 67 school reports were distributed and by 2017, this rose to 193. Reports feedback schools’ data, with national data for comparison, and aim to encourage and provide a resource for evidence-informed health action planning in the priority areas chosen by the schools. Schools have utilised the reports for different purposes; for example, teachers have used them for curriculum planning, student councils have used them for health action prioritisation, and school senior managers have used them as evidence in school inspections. The network manager identifies innovative use of the reports and facilitates sharing with other schools through the network’s termly newsletter and events for schools. These annual, face-to-face events allow network researchers to build relationships with schools, for schools to network with each other around their health and wellbeing work and for them to inform the strategic direction of the network from the ‘chalk-face’. This has led to schools identifying emerging priority policy areas for SHRN to address such as suicide and self harm and to work together on particular topics of concern, identified through their report data. Network progress is demonstrated by the fact that in 2013, one national event was organised attracting 22 attendees; in 2015, this rose to five events and 106 attendees, with the current cycle providing six national events with 109 attendees currently confirmed.

To strengthen engagement at the regional level, a local authority feedback report was piloted in cycle two to be distributed to all local authorities in cycle three. The report pools data from all schools in the local authority area and provides a resource for local authority staff and their partners for evidence-informed health planning. The report also has potential to support local authorities to meet their statutory duties under the Well-being of Future Generations (Wales) Act 2015. In the current cycle, networking events have also been expanded to focus on regional stakeholders, as well as seven additional engagement events with school pupils.

The network also utilises engagement activities that span different levels of the school health system in order to establish new social networks and interactions. Webinars and research briefs were initially designed for school engagement and knowledge exchange, but their value to others has been recognised and their reach extended to national and regional stakeholders. Webinars translate recent research findings, including analysis of the network surveys, in a format suited to a practice and policy-maker audience. In 2013, there was only one webinar, but in 2015, this rose to seven and in 2017, to date, three have been delivered. Network research is also translated for practitioner and policy-maker audiences via research briefs. These are succinct summaries of new research findings and to date, eight have been produced for schools and WNHSS staff. Both they and the webinars are also posted on the network website to be readily accessible to all the network’s collaborators. Finally, agents from all levels of the school system are encouraged to engage with each other around system change for health improvement through annual academic/practice seminars. These are a forum to undertake the co-production activities that are crucial for system self-regulation.

## Co-Production Activities for Establishing Norms and System Self-Regulation

The ‘Resource Investment and Linkage for New Social Agents and Resources’ section discussed the processes by which SHRN has developed capacity for research and evidence-informed practice at multiple levels of the system, enhancing the potential of network collaborators to be active agents and offering university resource to develop a self-regulated culture of evidence-informed prevention. This section describes how that capacity is utilised in co-producing research utilising a systems framework and standardised processes (Hawkins et al. 2017).

The network’s critical co-production activity is its Research Development Groups (RDGs), the vehicles through which transdisciplinary collaborations co-produce and develop research questions and complete research grant applications. Transdisciplinary teams also deliver successful grants and analyse the data from SHRN’s data infrastructure, which subsequently informs the policy and practice evidence base.

Network seminars, webinars, and Student Health and Wellbeing Reports facilitate the identification of potential RDG topics and membership. Network resources are dedicated to facilitating and leading RDGs, and processes are aligned with improving capacity development through the nurturing of early career researchers. In addition, the structure of RDGs provides an effective team-working mechanism where one person leads but is supported by others through peer review. This helps to alter social networks to facilitate information flow and collaboration across activity settings (Hawe 2015; Hawe et al. 2009).

A mature RDG registration process is in place and a SHRN academic Partnership Board is responsible for approving new RDGs. A Research Ideas and Development Group co-ordinates the resources and capacity dedicated to each RDG and brings together network-funded staff, and knowledge exchange, policy, practice and public involvement colleagues to discuss new opportunities and ideas for future development.

Involving policymakers in the co-production process has been linked to more efficient transfer of knowledge into practice (Oliver et al. 2014). Co-producing research with academics, policymakers and practitioners also helps theorise contextual conditions and addresses realist questions about what works, for whom, and under what circumstances (Fletcher et al. 2016). Within the RDG process, there is shared understanding of the importance of early developmental work to understand variations in local contexts and how this is critical for subsequent scaling up (Hawkins et al. 2016).

In this way, the RDG approach moves beyond a traditional research dissemination pathway, which begins with study results and then dissemination via publications. Instead, it emphasises cyclical pathways between research and policy and practice, with each study contributing to the general field of knowledge, which then creates feedback loops to influence future research (Newson et al. 2015).

To date, SHRN has been highly effective in coproducing research studies, with five RDGs in cycle one leading to two funded studies, rising to 56 RDGs and 32 funded studies in cycle three. These have ranged across intervention development, pilot and effectiveness studies and natural experiments. Depending on results, this provides significant potential for promoting evidence-based prevention programmes in future SHRN cycles.

## Reciprocal Outputs for Feedback Loops and System Stability

The final stage in the SHRN T-CAS network cycle is generating reciprocal outputs, which create system feedback loops. These can either reinforce and stabilise changes in the system or prevent new innovations from becoming embedded into system functioning. The network’s data infrastructure underpins much of this stage by generating outputs that support the aims and agendas of all the strategic partners, thereby strengthening the partnerships for network sustainability and continuation of the network cycle. The data infrastructure consists of biennial student health and school environment surveys conducted in all network schools. Data are shared directly with schools, local and national government for health action planning and monitoring. The value of providing such data that addresses the policy priorities of strategic partners and links health and educational activities cannot be underestimated in strengthening partnerships and promoting systems change.

At the school level, the data infrastructure generates Student Health and Wellbeing Reports (see ‘Network development to build new social networks and interactions’ section above), which inform local level action. Report content is guided by practice and school health needs identified through network events and by the current cycle the number of reports distributed had risen from 67 to 193.

At the regional level, feedback reports have been provided for all 21 local authorities for the first time in the current cycle, these, along with biennial and WNHSS environment reports, support staff to monitor health improvement activity and student wellbeing in their jurisdictions, identify and prioritise issues and evaluate their local health improvement actions, thus generating feedback loops to affect practice. The capacity development activities and the knowledge translation tools (webinars and research briefs) described previously build skills and knowledge among WNHSS for such evidence-informed practice.

At the population level, the data infrastructure provides partners with a national health surveillance and monitoring system which provides timely data on fast moving issues such as electronic cigarette uptake (de Lacy et al. 2017) and on vulnerable groups (Long et al. 2017). Related to this, the infrastructure’s flexibility means policy-relevant data can readily be collected and used to monitor new policies, such as the new curriculum. In the current cycle, the emergent system has been considerably strengthened by network provision of data for a range of national and regional indicators covering Welsh Government’s Children and Young People’s Wellbeing Monitor for Wales, Well-being of Future Generations Act indicators and Active Healthy Kids Wales Report Card. Whilst 21 regional reports are being developed for Well-being of Future Generations Act indicators. Of even greater value is the potential to use the infrastructure to conduct natural experiments of new policies, at little or no cost (Moore et al. 2017), to support policy-maker decision-making on maintaining or adapting policies.

Academics also benefit from the data infrastructure and schools’ engagement with the network. Student survey data directly identifies health and wellbeing issues facing the whole population and sub-groups, such as young people in care or those disengaged from school. The school environment survey provides context data to use in understanding relationships between school health policy and practice and student health, which can feed into intervention development. Data from the surveys is also used for purposive sampling for research projects. High levels of school engagement have also helped the network create a cadre of ‘research-ready’ schools, which is evidenced by how much easier recruitment of schools to new studies has become. This has been seen in a number of SHRN-adopted studies, positioned at different points in the MRC’s framework for developing and evaluating complex interventions (Craig et al. 2008), from intervention development and piloting (Hawkins et al. 2016) to the evaluation of effectiveness through cluster-randomised trials (Kidger et al. 2016). Schools have also participated in PhD projects investigating different processes within the network, such as use of Student Health and Wellbeing Reports within school complex systems and the school health system’s current capacity for evidence-informed practice. Findings from these will further refine the network’s reciprocal outputs to fuel the feedback loops that will keep the cycle turning.

## Discussion

SHRN represents a developing T-CAS network cycle approach to facilitate systems change towards a culture of prevention for health and well being. Whilst acknowledging CAS change processes can apply across the cycle, this paper has theorised and evidenced a clear sequential approach to address dominant CAS mechanisms at each developmental cycle. It should be noted that reorienting a national education system is challenging and likely to take numerous cycles to achieve. Given that a CAS, by its very nature, is complex and adaptive, it is not possible to approach system change with a priori indicators of success. However, a system change theory does identify activities and facilitates an understanding of the development of and inter relationship of system change processes across cycles. In this way, we have seen SHRN successfully embed itself in the system, both horizontally (across health, education and social care) and vertically (national, regional and schools) through new partnership working. These partnerships have led to multi-sector investment in the network across the cycles. This has secured system sustainability and is in itself a marker of its perceived value across health, education and social care at the highest level in government. The fact that SHRN has recruited every secondary school and engaged over 60% of the pupil population in Wales means that a new system structure has been successfully implemented within only three diffusion cycles.

SHRN has also had impacts in re-orientating the system to one of evidence generation. To date, 56 research studies have been co-produced and supported. Significant progress has been made in research capacity and network development to achieve this. The system has now also been re-orientated towards data-led practice opportunities. National, regional and school reporting systems have been newly established, and stakeholders have been presented with the potential to engage in data-led practice. Future cycles will need to focus on the development of school-based agents of change, to capitalise on such data-led practice opportunities in order to facilitate longer-term population health benefits.

These findings provide an understanding of the relationship between T-CAS activities and how SHRN has developed across its initial three cycles. In this, this paper has identified the key activities and processes to be examined in future empirical evaluation in order to shed further light on the mechanisms of action for effective system change. However, in examining the success of SHRN in laying the groundwork for systems change, elements of the Welsh context that might limit transferability should be acknowledged. The first of these relates to system events, which provide opportunities for strategic collaboration and system reorientation. Undoubtedly, recent policy developments in Wales represent significant changes to health and education policy and provide a fertile ground for promoting health. These policies could be seen to represent disruption points, whether SHRN existed or not. However, SHRN utilised changes in policy direction through consultation responses and partnership working to ensure that their implementation and evaluation was conducted with a transdisciplinary approach. New policies represent open doors for promoting health, but their success depends on strategic partnerships, which work to reorient the system towards including SHRN in its every day functioning.

The support of DECIPHer, with its strong links to policy and practice to promote rigorous pragmatic evaluations of complex interventions, has also been key. Furthermore, SHRN developed out of another research network, PHIRN, funded by Welsh Government from 2005. PHIRN focuses on developing capacity in transdisciplinary research methodologists for policy development and evaluation. The provision of a supportive multidisciplinary research centre and a pool of methodologists provided a historical bed of successful policy and practice partnerships and readily available social agents to affect systems change opportunities. In this sense, SHRN benefited from the systems disruption begun first by PHIRN and then DECIPHer, which helped to shape system evolution and reorganisation over a 10-year period. A systems intervention like SHRN can therefore be conceptualised as requiring much longer periods to effect change, and at the very least that capacity development is required at early stages of the network cycle. This would allow transdisciplinary social agents to exploit disruptions to system functioning to promote intervention assimilation through system reorganisation.

Whilst acknowledging that historical and national policy developments in Wales created a supportive context for systems change, other elements of T-CAS networks are more readily transferable across contexts. The first of these is the value of a phased diffusion approach to implementation. This limits the risk of networks ‘washing out’ as self-organisation processes work to maintain the status quo and increase the likelihood of new practice becoming gradually embedded within system functioning (Hawe 2015; Hawe et al. 2009). Gradual embedding may increase the chance of coherent emergent outcomes, e.g. advances in health improvement practice at different levels of the system through working with the system to achieve change and reducing uncertainty (Hawe 2015; Hawe et al. 2009).

SHRN’s three-phase approach first examined feasibility, then scalability and finally total system diffusion over a 6-year period (2013–2018). Such an approach recognises the importance of establishing and stabilising emergent iterations through feedback loops for system continuation. Investment from the MRC supported a feasibility and scoping phase, during which the network was launched, school engagement strategies were refined and the Advisory Board was established. Key tasks were to assess the acceptability of new systems and structures and identify shared agendas and collaborative activities to establish stage one of the T-CAS network cycle.

A phased approach also addressed a key obstacle in this area, namely the lack of an established funding structure for network development and maintenance outside Clinical Research. Such an approach allowed a coalition of funders to be built slowly by identifying the resources needed and through successful implementation and the delivery of outputs. The pilot phase was supported by research council funding and relied on in kind partner contributions for value-added activity. This first cycle facilitated system change process and provided an important foothold for SHRN. Health and Care Research Wales and Public Health Wales provided funding to assess the resources and structures required for network scale up and extended the network’s research, engagement and capacity development activities to embed the network further. This not only supported an evidence-based scale up but also secured investment from both health and education Cabinet Secretaries in Welsh Government. In the absence of traditional funding structures, the Network is reliant on such investment for maintaining scale and sustainability.

The second element concerns the active ingredients of the network that have been instrumental in triggering changes in the CAS towards laying the groundwork for a culture of health improvement. The first of these concerns the nature and focus of strategic partnerships. SHRN’s development of hierarchical and horizontal partnerships, both within SHRN and across the national policy landscape recognises the need to engage across systems, as well as with nested sub-systems and supra-systems to promote complex systems change (Keshavarz et al. 2010). It is also useful to reflect, that the development of mutual agendas is more readily facilitated by identifying and developing common policy areas that link these sectors and disciplines. In this way, the network becomes an active agent in creating a supportive system, with academics taking on both research and lobbying roles. Mutual agendas provide an operational framework for collaboration and securing resources for systems change. It is important to recognise that frequently, these resources can be drawn from those already within the system, with partnerships and, in particular, transdisciplinary network spanning roles promoting integration for value-added activity (Burt 2004).

Perhaps the most important lesson for transferability is the value of a T-CAS network cycle approach and the principles and processes that support a co-production approach with stakeholders. It is here that systems are self-regulated as new norms are adopted, and the system may move towards a new attractor state. The fact that SHRN identified and developed reciprocal benefits for all partners clearly supported this. Finally, although SHRN benefited from a ready supply of research methodologists as social change agents, the need for school workforce development in evidence informed practice is likely to be found across many educational contexts internationally. It is here perhaps in developments to initial teacher training and continuous professional development that the greatest challenges lie in developing fertile ground for a culture of prevention.
